# Supplementary material for: Risk of death following COVID-19 vaccination or positive SARS-CoV-2 test in young people in England
Source: Nat Commun. 2023 Mar 27;14:1541. doi: 10.1038/s41467-023-36494-0 (PMC10043280; doi:10.1038/s41467-023-36494-0)
Supplement: Supplementary file 1 — Supplementary Information [file 41467_2023_36494_MOESM1_ESM.pdf]

Risk of death following COVID-19 vaccination or positive SARS-CoV-2 test in young people in England

Supplementary Information

Supplementary Fig. 1 | Sample flow for the death registrations and Hospital Episode Statistics datasets. a Deaths since vaccination. **b** Deaths since positive test for SARS-CoV-2.

a

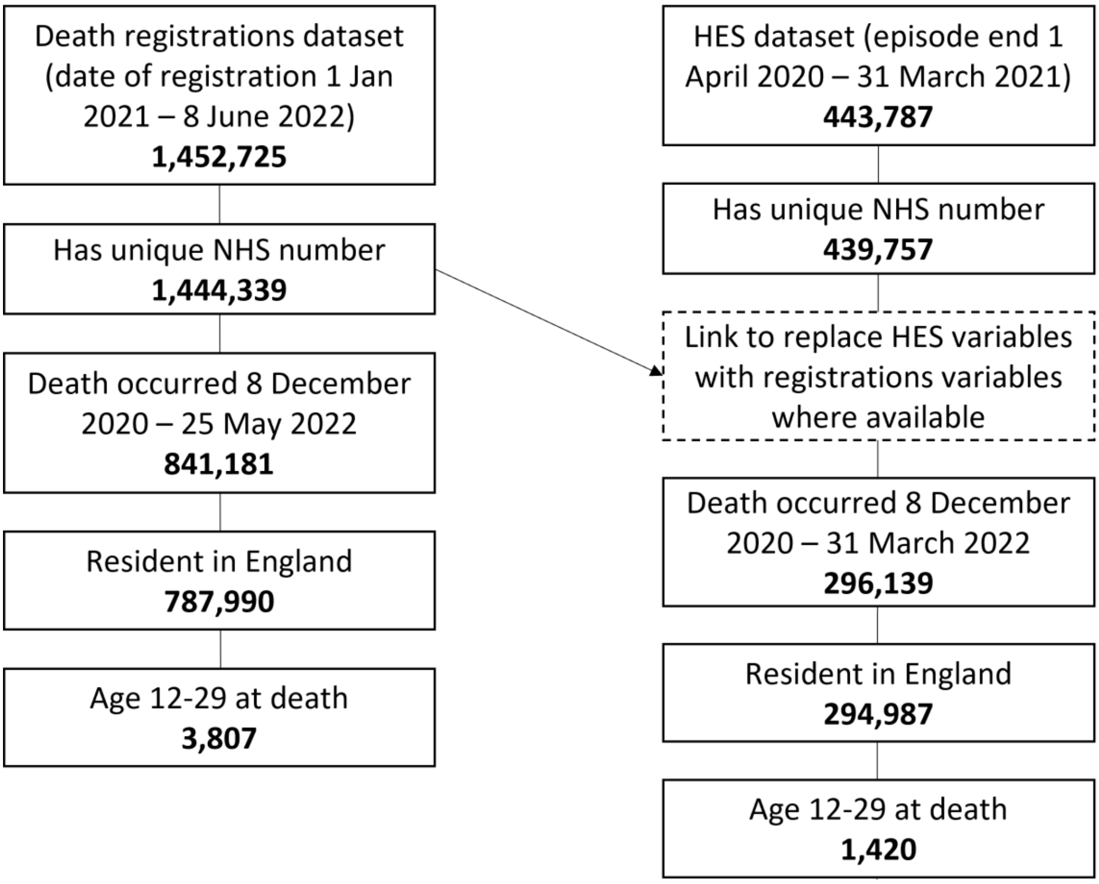

b

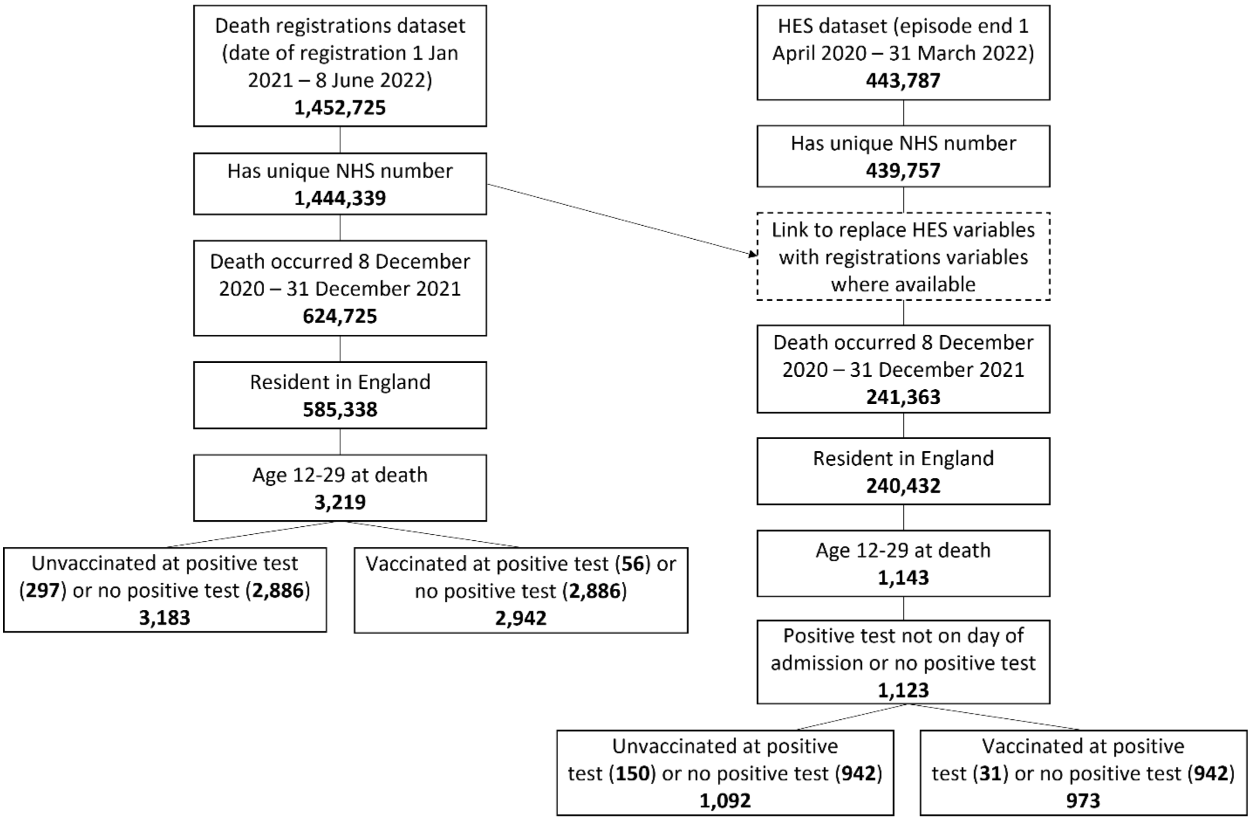

Death registrations dataset (date of registration 1 Jan 2021 – 8 June 2022)
1,452,725
Has unique NHS number
1,444,339
Death occurred 8 December 2020 – 31 December 2021
624,725
Resident in England
585,338
Age 12-29 at death
3,219
Unvaccinated at positive test (297) or no positive test (2,886)
3,183
Vaccinated at positive test (56) or no positive test (2,886)
2,942
HES dataset (episode end 1 April 2020 – 31 March 2022)
443,787
Has unique NHS number
439,757
Link to replace HES variables with registrations variables where available
Death occurred 8 December 2020 – 31 December 2021
241,363
Resident in England
240,432
Age 12-29 at death
1,143
Positive test not on day of admission or no positive test
1,123
Unvaccinated at positive test (150) or no positive test (942)
1,092
Vaccinated at positive test (31) or no positive test (942)
973

Supplementary Fig. 2 | Number of deaths that occurred each week for weeks since December 8 2020, young people aged 12-29 on date of death, by last vaccination dose received. All-cause and cardiac deaths are shown for the ONS death registration data, and all-cause deaths are shown for the HES data. Vaccination status was suppressed when the number of deaths were small and could risk being disclosive.

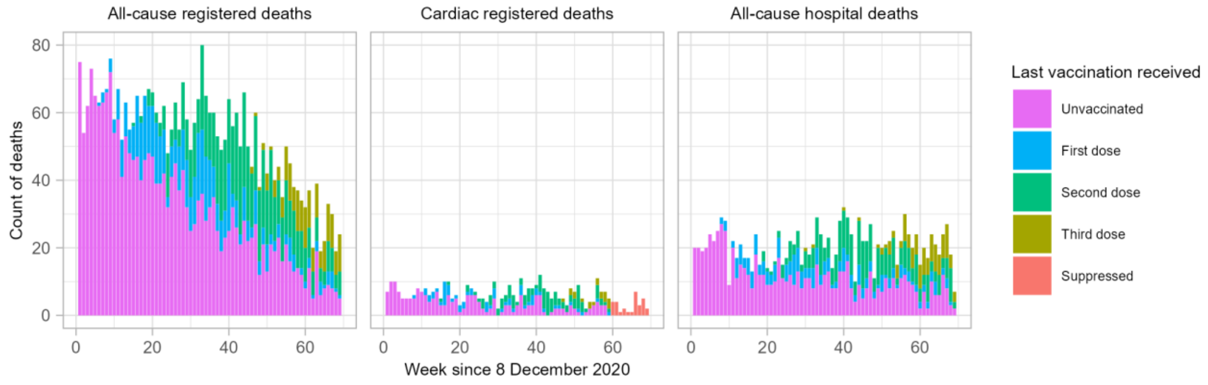

Supplementary Fig. 3 | Number of deaths that each week for weeks since December 8 2020, for deaths recorded by 31 December, young people aged 12-29 on date of death, by whether they had had a positive test for SARS-CoV-2 at any point. All-cause and cardiac deaths are shown for the ONS registrations data, and all-cause deaths are shown for the HES data. Positive SARS-CoV-2 status was suppressed when the number of deaths were small and could risk being disclosive.

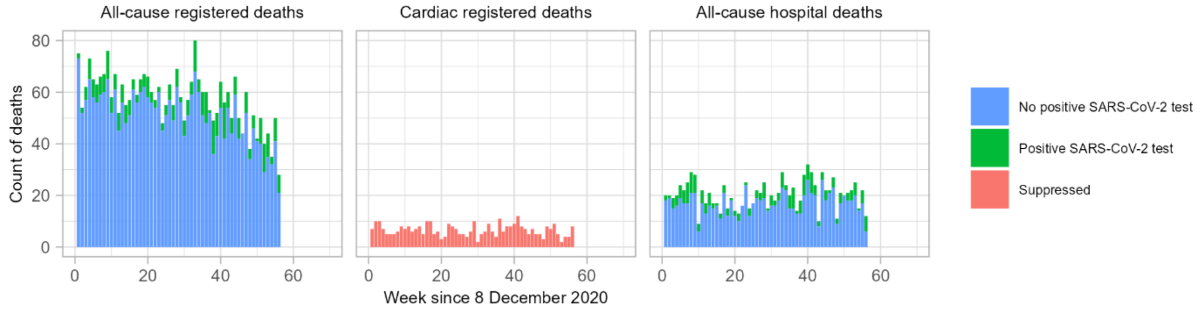

Supplementary Fig. 4 | Number of deaths each week since vaccination (by dose) and positive test for SARS-CoV-2for the registrations (all-cause and cardiac deaths) and HES (all-cause deaths) data. **a** Deaths since vaccination. **b** Deaths since positive test for SARS-CoV-2.

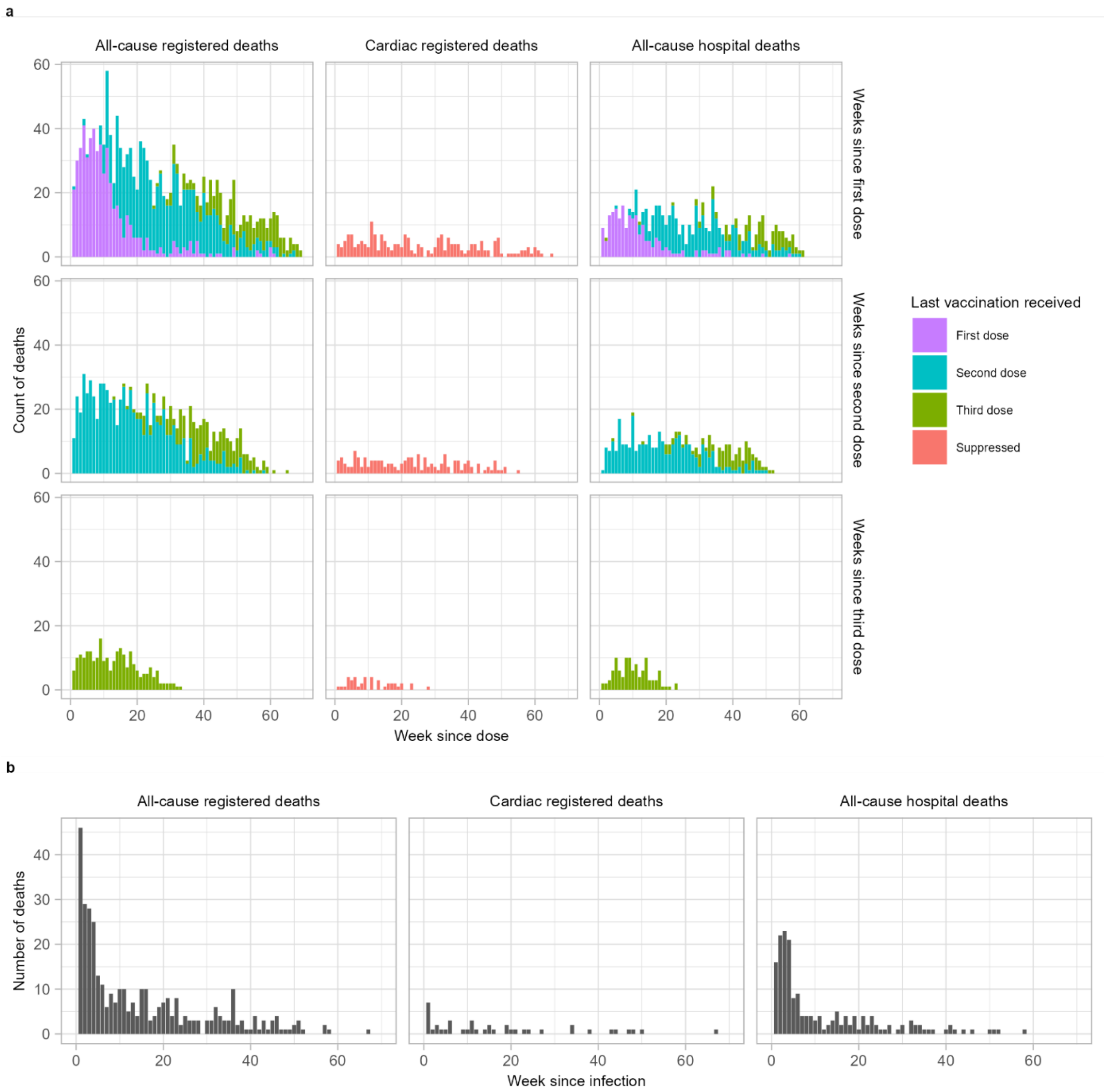

Supplementary Fig. 5 | Relative incidence of cardiac death and all-cause death in the risk period after vaccination compared to the baseline period, broken down by sex and vaccine vector. Data are presented as incidence rate ratio with 95% confidence intervals. n=3,807 all-cause registered deaths, 444 cardiac registered deaths and 1,420 all-cause hospital deaths. **a** Results for males **b** Results for females.

a

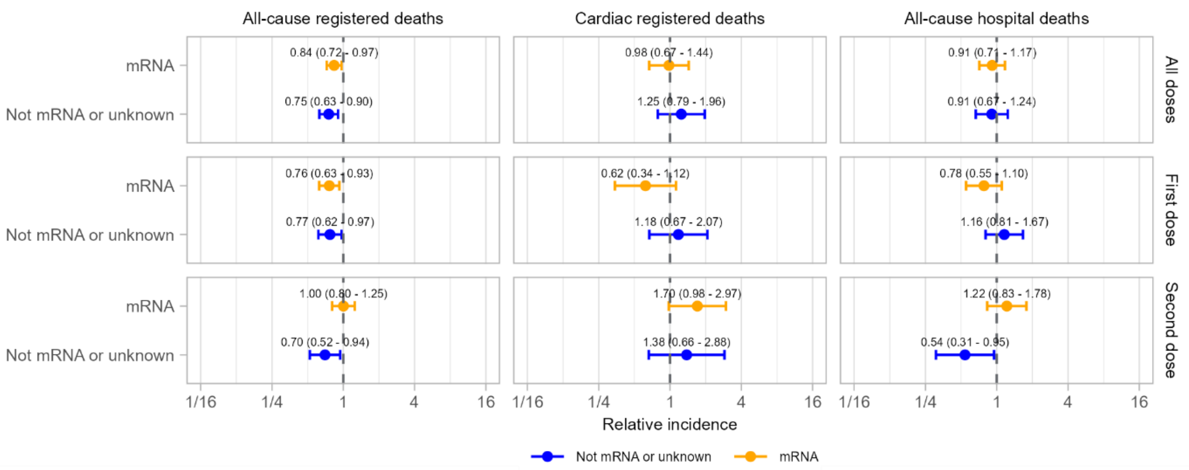

b

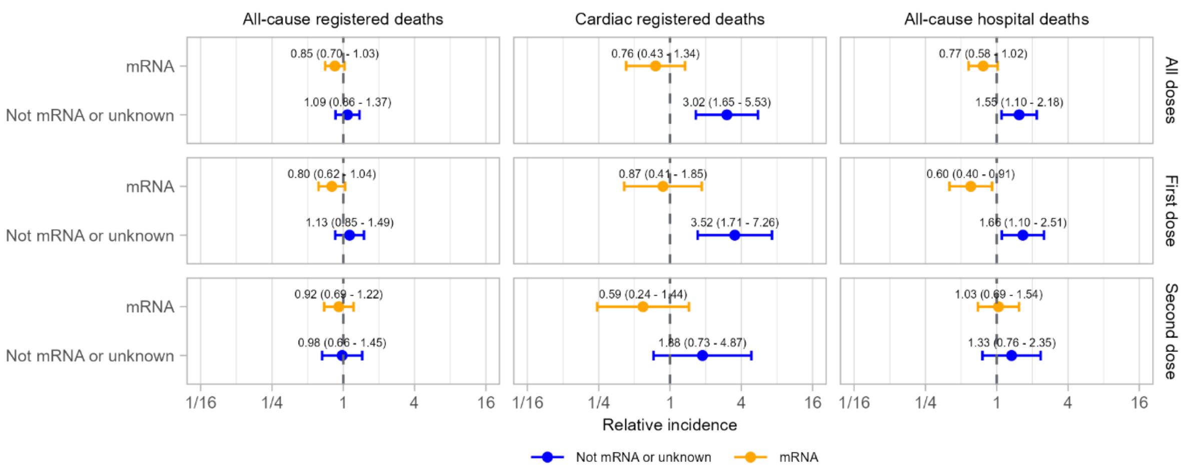

Supplementary Fig. 6 | Relative incidence of cardiac deaths after a second dose of an mRNA vaccination for males for different lengths of risk period. Data are presented as incidence rate ratio with 95% confidence intervals. n=444 cardiac registered deaths. Week 12 is indicated in orange.

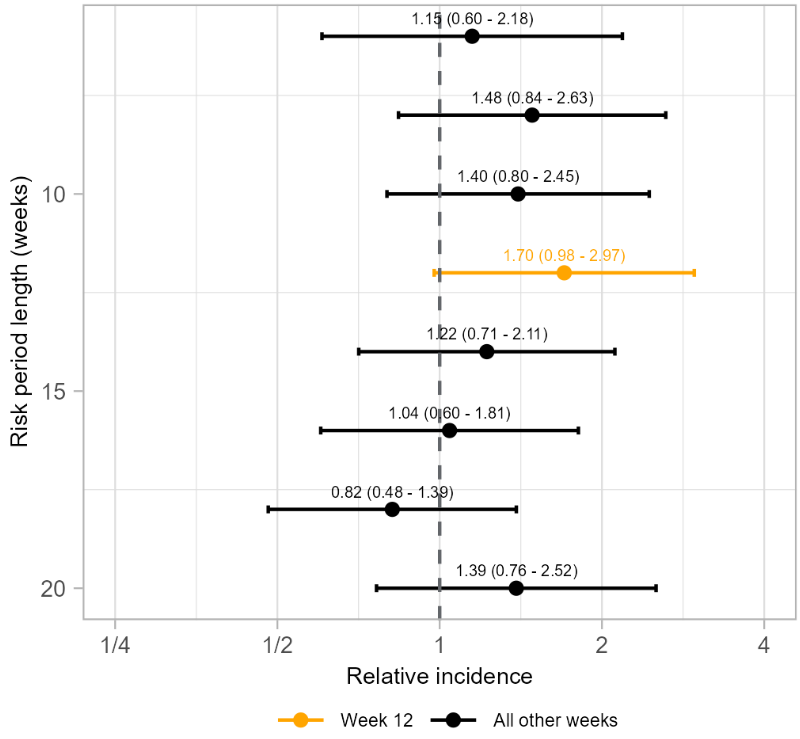

Supplementary Fig. 7 | Relative incidence of cardiac and all-cause deaths after vaccination for different lengths of risk period. Week 12 is indicated in orange. Risk periods exceeding the interval between doses are subject to negative bias (the median interval was 10 weeks between dose 1 and 2 and 27.5 weeks between dose 2 and 3). Models are adjusted for calendar time. Data are presented as incidence rate ratio with 95% confidence intervals. n=3,807 all-cause registered deaths, 444 cardiac registered deaths and 1,420 all-cause hospital deaths. **a** All doses combined. **b** Separate doses.

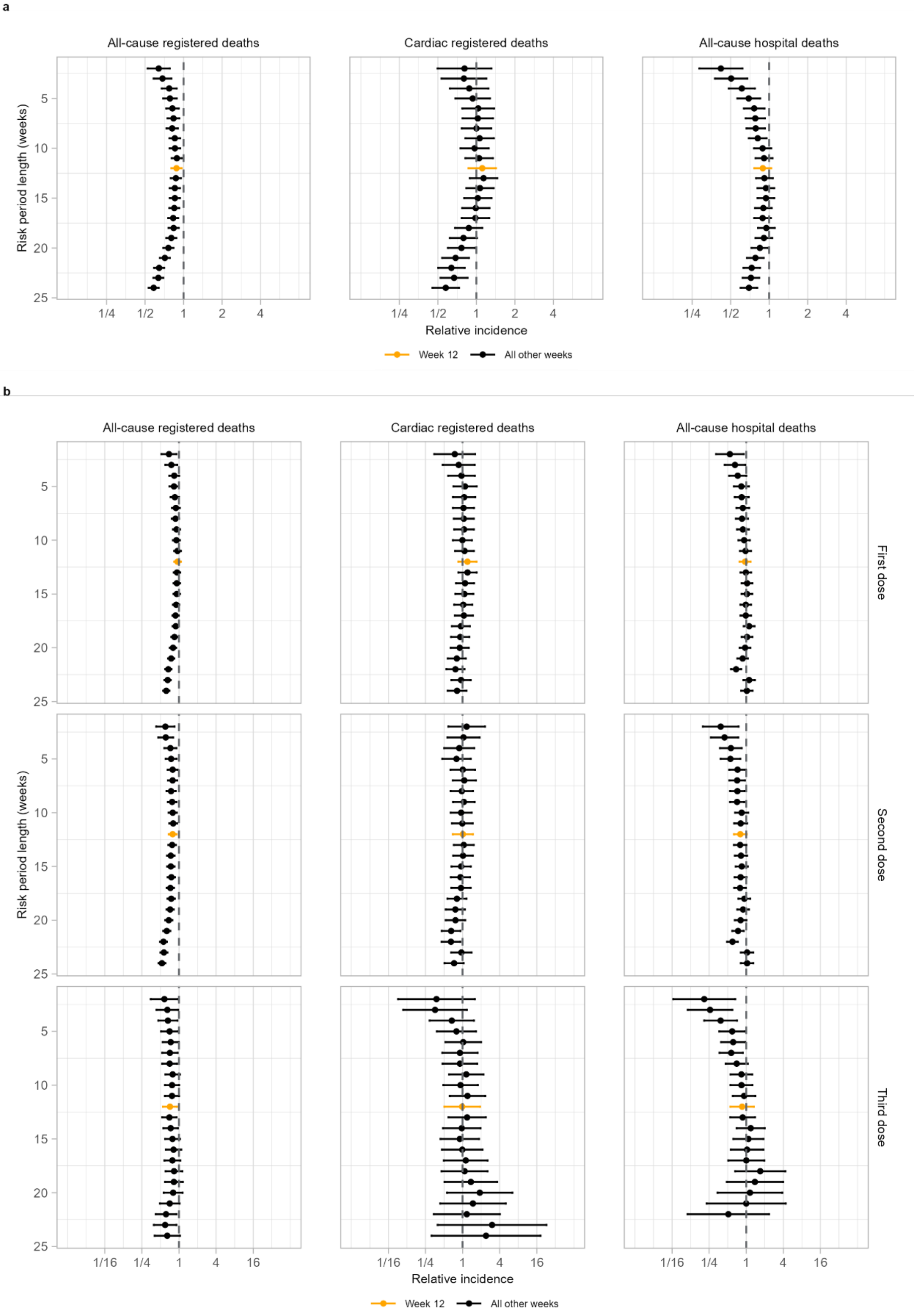

Supplementary Fig. 8 | Relative incidence of cardiac and all-cause deaths after positive SARS-CoV-2 test, for different lengths of risk period. Data are presented as incidence rate ratio with 95% confidence intervals. n=3,219 all-cause registered deaths, 369 cardiac registered deaths and 1,123 all-cause hospital deaths. **a** Unvaccinated at positive test for SARS-CoV-2. **b** Vaccinated at positive test for SARS-CoV-2. Cardiac registered deaths not shown due to small numbers.

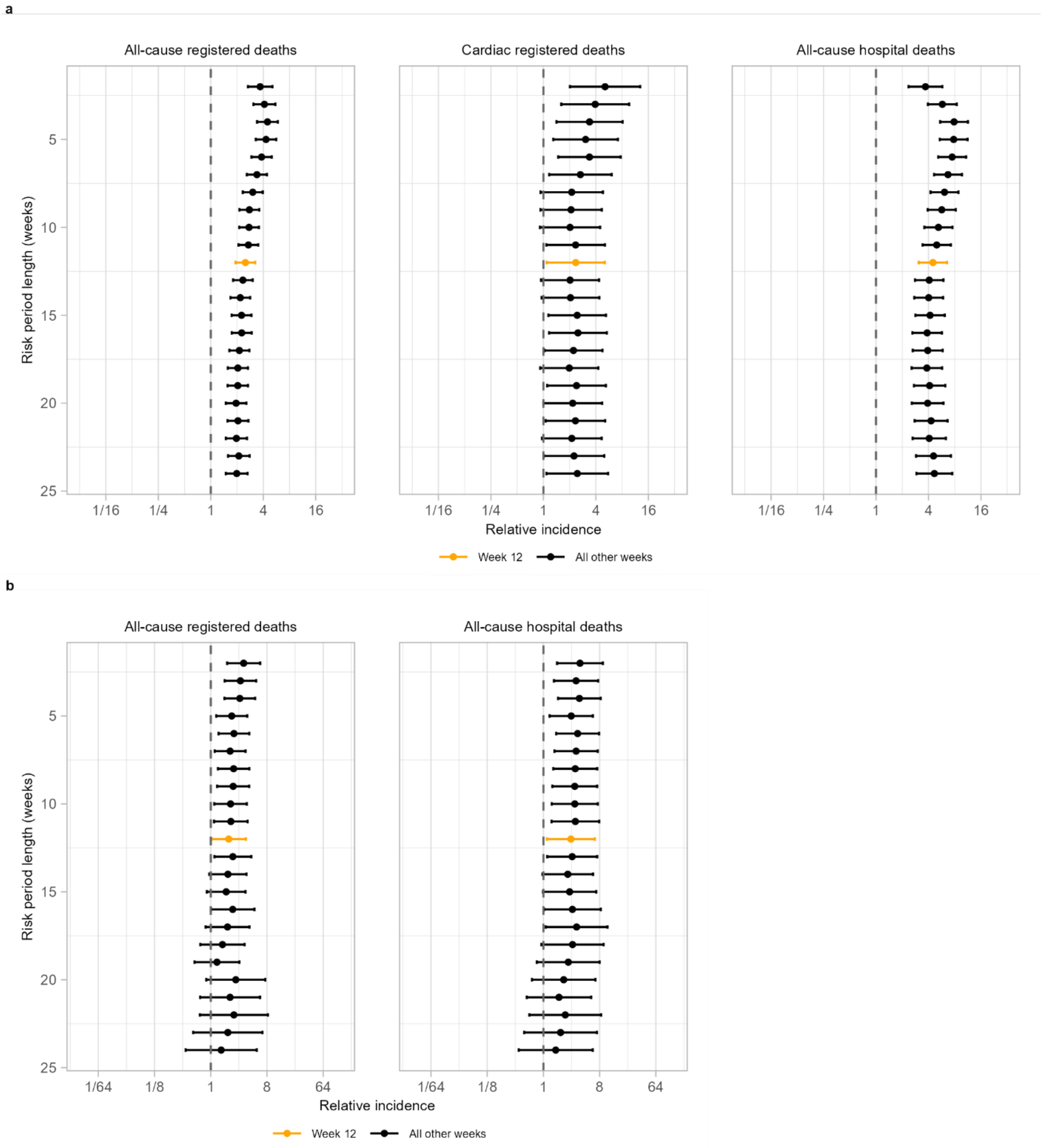

Supplementary Fig. 9 | Relative incidence of cardiac death and all-cause death in each of the 12 weeks in the risk period after vaccination and in the risk period as a whole (1-12 weeks), compared to the baseline period. Calendar time is adjusted fortnightly intervals. Data are presented as incidence rate ratio with 95% confidence intervals. n=3,807 all-cause registered deaths, 444 cardiac registered deaths and 1,420 all-cause hospital deaths. **a** Results for all doses combined. **b** Results by dose.

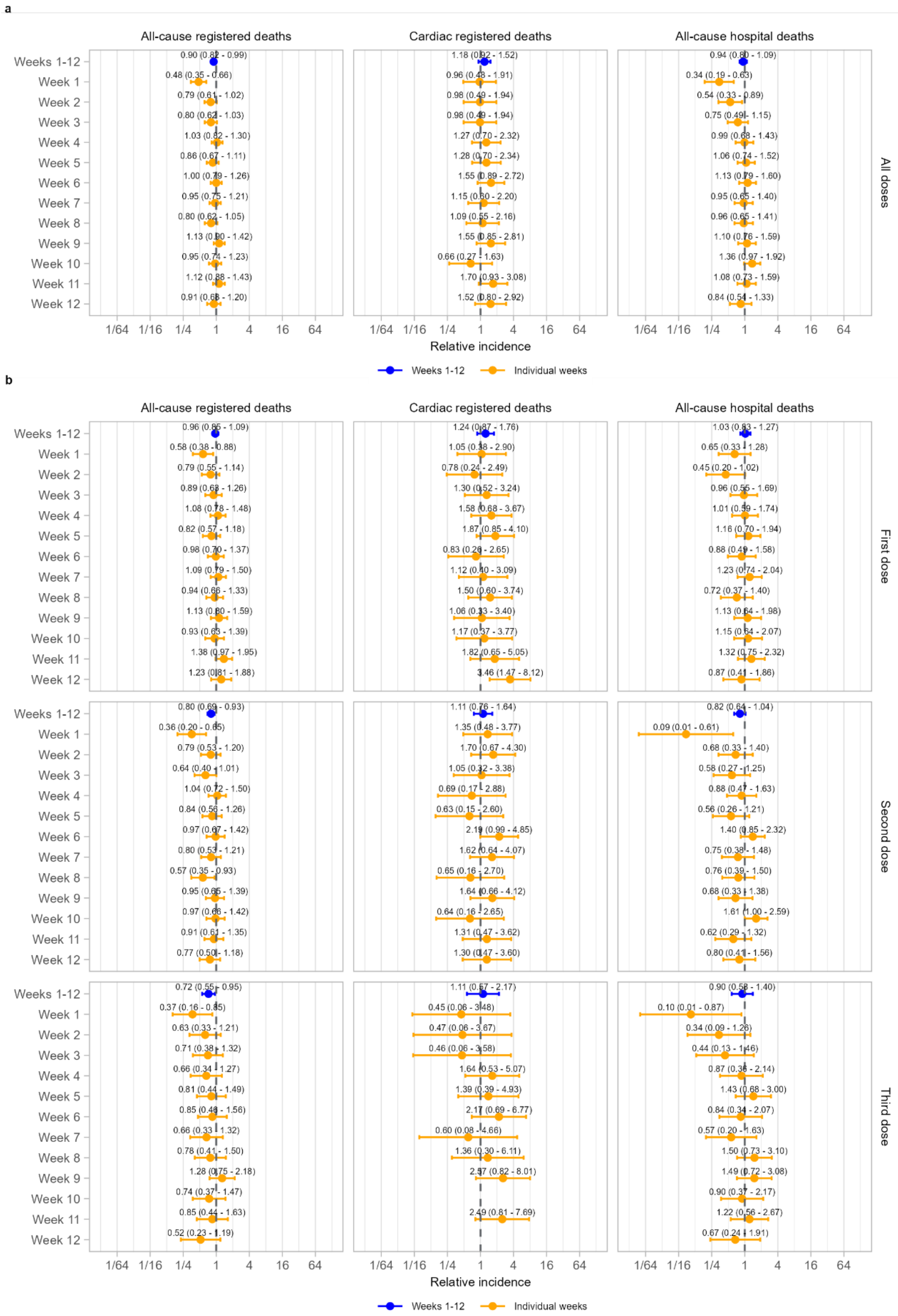

Supplementary Fig. 10 | Relative incidence of cardiac death and all-cause death in each of the 12 weeks in the risk period after positive test for SARS-CoV-2 and in the risk period as a whole, compared to the baseline period. Calendar time is adjusted fortnightly intervals. Data are presented as incidence rate ratio with 95% confidence intervals. n=3,219 all-cause registered deaths, 369 cardiac registered deaths and 1,123 all-cause hospital deaths. **a** Results for individuals unvaccinated at time of positive test. **b** Results for individuals vaccinated at time of positive test. Cardiac registered deaths not shown due to small numbers.

a

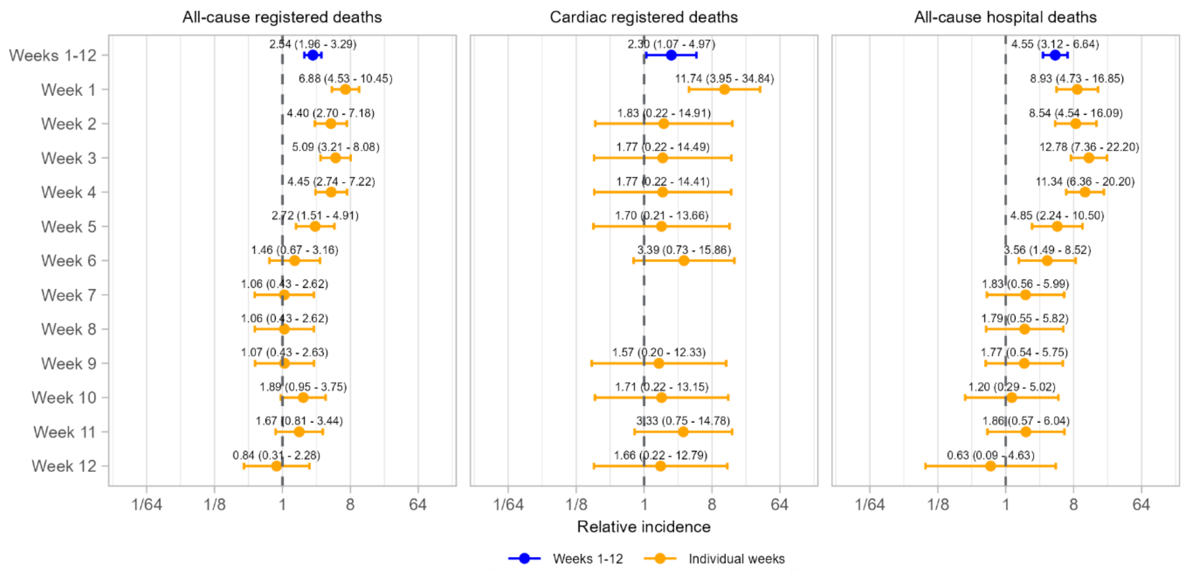

b

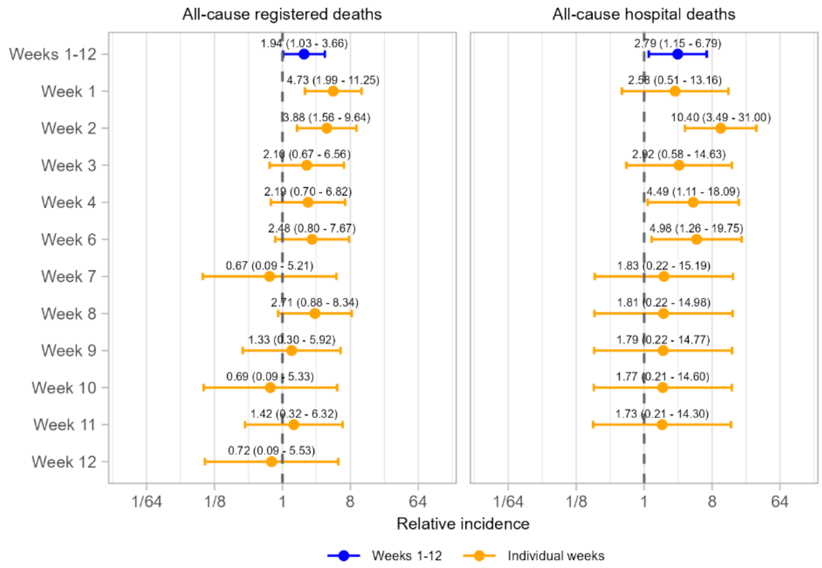

Supplementary Fig. 11 | Relative incidence of all-cause hospital death in each of the 12 weeks in the risk period after vaccination and in the risk period as a whole (1-12 weeks), compared to the baseline period. Individuals with a SARS-CoV-2 positive result on the day of hospital admission are excluded. Data are presented as incidence rate ratio with 95% confidence intervals. n=1,420 all-cause hospital deaths.

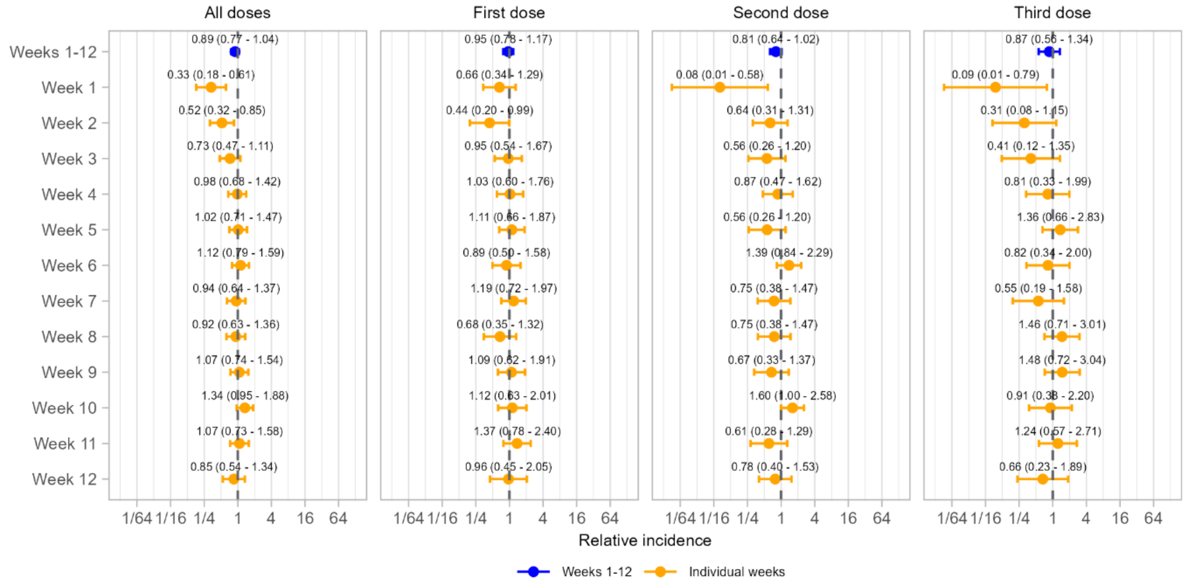

Supplementary Fig. 12 | Relative incidence of all-cause hospital death in each of the 12 weeks in the risk period after positive test for SARS-CoV-2 and in the risk period as a whole (1-12 weeks), compared to the baseline period. Individuals with a SARS-CoV-2 positive result on the day of hospital admission are included. Data are presented as incidence rate ratio with 95% confidence intervals. n=1,123 all-cause hospital deaths. **a** Results for individuals unvaccinated at time of positive test. **b** Results for individuals vaccinated at time of positive test.

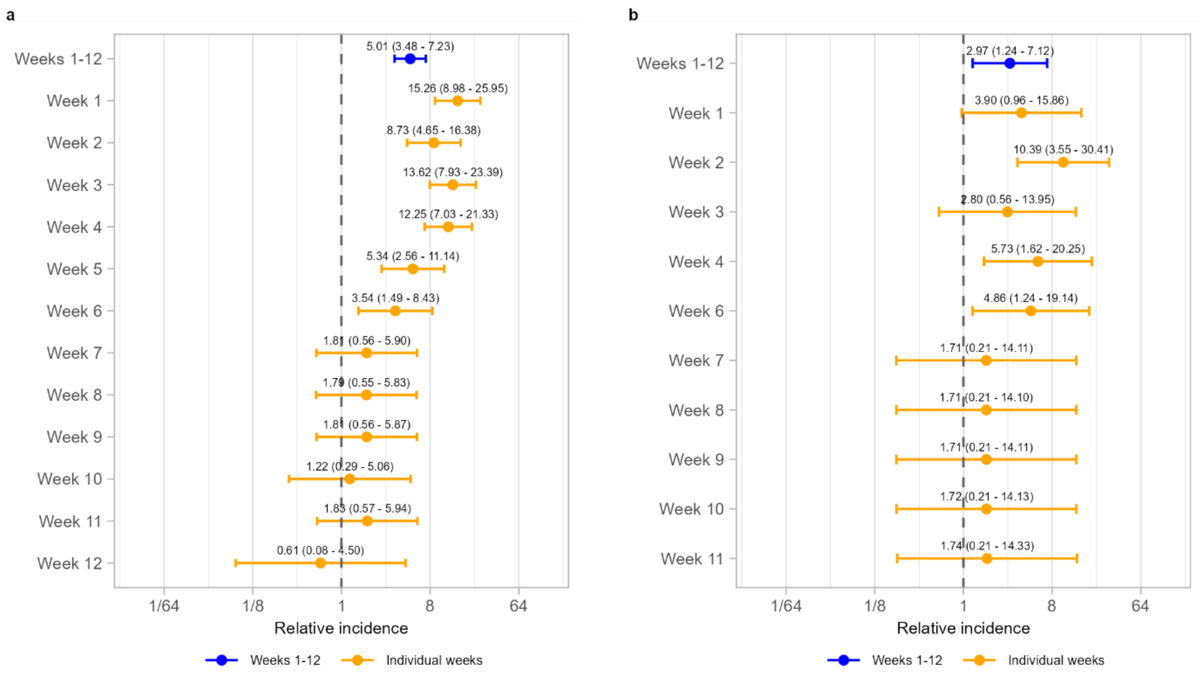

Supplementary Table 1 | Vaccine vectors by dose for the death registrations dataset for the first and second doses. 'mRNA' includes the Pfizer BNT162b2 Pfizer-BioNTech and mRNA-1273 Moderna vaccines. 'Not mRNA or unknown' includes the ChAdOx1 Oxford-AstraZeneca vaccine, all other recorded vaccine manufacturers and non-recorded vaccination manufacturers. 239 out of 242 third doses in the registrations dataset, and 108 out of 110 third doses in the hospital dataset were mRNA based.

First dose	Second dose	Number of individuals in registrations dataset	Number of individuals in hospital dataset
mRNA	mRNA	505	228
mRNA	Not received	334	120
mRNA	Not mRNA or unknown	111	51
Not received	Not received	2,295	790
Not mRNA or unknown	Not received	199	84
Not mRNA or unknown	Not mRNA or unknown	363	147

Supplementary Table 2 | Number of individuals vaccinated who received at least one of each vaccine vector type, by sex.

	Male			Female		
	All-cause registered deaths	Cardiac registered deaths	Hospital registered deaths	All-cause registered deaths	Cardiac registered deaths	Hospital registered deaths
Vaccinated with at least one dose of mRNA	589	82	222	361	44	177
Vaccinated with at least one dose of non mRNA or unknown	416	66	157	257	41	126

Supplementary Table 3 | Number of individuals vaccinated with each vaccine vector type, by time period vaccinated for the first and second doses for the registrations dataset.

	Until 7 April 2021	After 7 April 2021
First dose of non-mRNA or unknown	541	20
Second dose of non-mRNA or unknown	28	446
First dose of mRNA	424	522
Second dose of mRNA	54	451

Supplementary Methods 1 | Calculations of absolute effects

We derived estimates of absolute effects using an established method for the self-controlled case series design [40].

Between 9 September 2021 and 31 December 2021, there were 1,131,503 unvaccinated people who tested positive for SARS-CoV-2, and 1,105,761 vaccinated individuals. There were 158 deaths within 12 weeks of a positive SARS-CoV-2 test in unvaccinated individuals, 41 in those who were vaccinated.

Between 8 December 2020 and 25 May 2022, there were 4,012,520 females aged 12 to 29 who received at least one dose of a COVID-19 vaccine between these dates, of which 177,042 received a first dose of a non mRNA or unknown vaccine vector. There were 25 cardiac registered deaths of females within 12 weeks of receiving the first dose of a COVID-19 vaccination, of which 15 had received a first dose of a non mRNA or unknown vaccine vector.

Between 8 December 2020 and 25 May 2022, there were 2,958,893 men aged 12 to 29 who received a second dose of an mRNA based COVID-19 vaccine between these dates. There were 20 cardiac registered deaths of men within 12 weeks of receiving the second dose of an mRNA COVID-19 vaccination.
