# Supplementary material for: Risk of death following COVID-19 vaccination or positive SARS-CoV-2 test in young people in England
Source: Nat Commun. 2023 Mar 27;14:1541. doi: 10.1038/s41467-023-36494-0 (PMC10043280; doi:10.1038/s41467-023-36494-0)
Supplement: Supplementary file 3 — Reporting Summary [file 41467_2023_36494_MOESM3_ESM.pdf]

nature portfolio

Corresponding author(s): Charlotte Bermingham

Last updated by author(s): Feb 2, 2023

Reporting Summary

Nature Portfolio wishes to improve the reproducibility of the work that we publish. This form provides structure for consistency and transparency in reporting. For further information on Nature Portfolio policies, see our Editorial Policies and the Editorial Policy Checklist.

Statistics

For all statistical analyses, confirm that the following items are present in the figure legend, table legend, main text, or Methods section.

n/a	Confirmed	
☐	☒	The exact sample size (*n*) for each experimental group/condition, given as a discrete number and unit of measurement
☐	☒	A statement on whether measurements were taken from distinct samples or whether the same sample was measured repeatedly
☒	☐	The statistical test(s) used AND whether they are one- or two-sided<br>*Only common tests should be described solely by name; describe more complex techniques in the Methods section.*
☐	☒	A description of all covariates tested
☐	☒	A description of any assumptions or corrections, such as tests of normality and adjustment for multiple comparisons
☐	☒	A full description of the statistical parameters including central tendency (e.g. means) or other basic estimates (e.g. regression coefficient) AND variation (e.g. standard deviation) or associated estimates of uncertainty (e.g. confidence intervals)
☒	☐	For null hypothesis testing, the test statistic (e.g. *F*, *t*, *r*) with confidence intervals, effect sizes, degrees of freedom and *P* value noted<br>*Give P values as exact values whenever suitable.*
☒	☐	For Bayesian analysis, information on the choice of priors and Markov chain Monte Carlo settings
☒	☐	For hierarchical and complex designs, identification of the appropriate level for tests and full reporting of outcomes
☒	☐	Estimates of effect sizes (e.g. Cohen's *d*, Pearson's *r*), indicating how they were calculated

Our web collection on statistics for biologists contains articles on many of the points above.

Software and code

Policy information about availability of computer code

Data collection	Data linkage and preparation was conducted using R 3.5, Python version 3.6 and Spark version 2.4
Data analysis	All analyses were conducted using R 3.5.

For manuscripts utilizing custom algorithms or software that are central to the research but not yet described in published literature, software must be made available to editors and reviewers. We strongly encourage code deposition in a community repository (e.g. GitHub). See the Nature Portfolio guidelines for submitting code & software for further information.

Data

Policy information about availability of data

All manuscripts must include a data availability statement. This statement should provide the following information, where applicable:

- Accession codes, unique identifiers, or web links for publicly available datasets
- A description of any restrictions on data availability
- For clinical datasets or third party data, please ensure that the statement adheres to our policy

The source data used in this study is subject to controlled access due to its sensitive nature. The ONS is working to make death registration data linked to vaccination data from the National Immunisation Management Service available on the ONS Secure Research Service. Test and Trace data (unlinked) is also available through the SRS. Access is available to accredited researchers. Details of the application requirements and process, and the use of data, are available at ons.gov.uk/aboutus/whatwedo/statistics/requestingstatistics/secureresearchservice. Microdata on death and vaccination may also be accessed through the NHS-Digital Data

Access Request Service. Hospital Episode Statistics data is not available through the ONS; this data is held by NHS England. All statistical data used in this study are available from the Office for National Statistics website.

Human research participants

Policy information about studies involving human research participants and Sex and Gender in Research.

Reporting on sex and gender	No human research participants were involved in the research. Only administrative data sources were used. Where sex is reported, it refers to self-reported sex as recorded in the 2011 Census.
Population characteristics	No human research participants were involved in the research.
Recruitment	No human research participants were involved in the research.
Ethics oversight	No human research participants were involved in the research.

Note that full information on the approval of the study protocol must also be provided in the manuscript.

Field-specific reporting

Please select the one below that is the best fit for your research. If you are not sure, read the appropriate sections before making your selection.

☐ Life sciences ☒ Behavioural & social sciences ☐ Ecological, evolutionary & environmental sciences

For a reference copy of the document with all sections, see nature.com/documents/nr-reporting-summary-flat.pdf

Behavioural & social sciences study design

All studies must disclose on these points even when the disclosure is negative.

Study description

Self-controlled case series, qualitative data

Research sample

Individuals aged 12–29 years, resident in England, who died between 8 December 2020 and 25 May 2022, registered by 8 June 2022, and individuals aged 12-29 years who died in hospital recorded in Hospital Episode Statistics between 8 December 2020 and 31 March 2022. Data is national, linked electronic health record data in England. Data sources are Office for National Statistics death registrations, Hospital Episode Statistics, the National Immunisation Management Service and a supplementary extract of NHS point of care data for COVID-19 vaccinations and Test and Trace data on SARS-CoV-2 tests.

Only individuals who experienced the outcome - death - were included in the datasets due to the nature of the analysis technique used. The 12-29 age group the study sample was chosen in order to investigate the risk of death after vaccination and SARS-CoV-2 infection because serious side effects have been most commonly reported in this age group and the benefit of vaccination is lower than in older age groups, therefore the risks are particularly important to investigate. The study periods used were determined by the earliest vaccination administered (8 December 2020) and the latest data available for each dataset. The setting was restricted to England residents due to datasets available.

The data sample was representative of all deaths that occurred because population level datasets were used that included deaths of all individuals provided an NHS number was available for linkage, which covered 99.4% of the death registrations and 99.1% of the Hospital Episode Statistics deaths used.

For the death registrations based dataset, 66.3% of the deaths were male, 60.3% were unvaccinated, 14.0% had received one vaccination, 16.4% had received two vaccinations and 6.4% had received three vaccinations. For the analysis of infections, which included deaths registered up to 31 December 2021, 9.23% had a positive SARS-CoV-2 test and were unvaccinated at time of the test, 1.7% had a positive SARS-CoV-2 test and were vaccinated at the time of test, and the remaining 83.9% had no positive SARS-CoV-2 test.

For the Hospital Episode Statistics based dataset, 59.2% of the deaths were male, 55.6% were unvaccinated, 14.4% had received one vaccination, 22.3% had received two vaccinations and 7.8% had received three vaccinations. For the analysis of infections, which included deaths registered up to 31 December 2021 where the positive test was not on the day of hospital admission or there was no positive test, 13.4% had a positive SARS-CoV-2 test and were unvaccinated at time of the test, 2.8% had a positive SARS-CoV-2 test and were vaccinated at the time of test, and the remaining 83.9% had no positive SARS-CoV-2 test.

Sampling strategy

No sampling was needed. The full dataset was used for analysis. No qualitative data was used.

Data collection

Administrative data was used. Therefore the data was recorded in response to an event occurring e.g. death/vaccination and not necessarily for the purposes of research e.g. use of mortality data from death registration certificates and use of vaccination data from the National Immunisation Management System (NIMS) used to record vaccinations in the population to aid with the vaccinations roll-out. The researchers were not involved in the collection of any of the data used in the analysis.

Timing	Records were included of individuals who died between 8 December 2020 and 25 May 2022, 1 March 2022 or 31 December 2021 depending on the latest data available for the dataset used.
Data exclusions	For the analysis of vaccinations using death registrations, the following data exclusions were applied, in order, to the original dataset containing 1,452,725 death registrations from 1 Jan 2021 to 8 June 2022:<br>Individuals with no NHS number (8,386 exclusions), deaths that occurred before 8 December 2020 or after 25 May 2022 (603,158 exclusions), non residents of England (53,191 exclusions), aged less than 12 or 30+ (784,183 exclusions).<br><br>For the analysis of vaccinations using hospital episode statistics, the following data exclusions were applied, in order, to the original dataset containing 443,787 recorded hospital deaths from 1 April 2020 to 31 March 2021:<br>Individuals with no NHS number (4,030 exclusions), deaths that occurred before 8 December 2020 or after 31 March 2022 (143,618 exclusions), non residents of England (1,152 exclusions), aged less than 12 or 30+ (293,567 exclusions).<br><br>For the analysis of infections using death registrations, the following data exclusions were applied, in order, to the original dataset containing 1,452,725 death registrations from 1 Jan 2021 to 8 June 2022:<br>Individuals with no NHS number (8,386 exclusions), deaths that occurred before 8 December 2020 or after 31 December 2021 (819,614 exclusions), non residents of England (39,387 exclusions), aged less than 12 or 30+ (582,119 exclusions). For the analysis of infections of people unvaccinated at positive test, 56 people were were vaccinated at positive test were excluded. For the analysis of infections of people vaccinated at positive test, 297 people were were unvaccinated at positive test were excluded.<br><br>For the analysis of infections using hospital episode statistics, the following data exclusions were applied, in order, to the original dataset containing 443,787 recorded hospital deaths from 1 April 2020 to 31 March 2021:<br>Individuals with no NHS number (4,030 exclusions), deaths that occurred before 8 December 2020 or after 31 March 2022 (198,394 exclusions), non residents of England (931 exclusions), aged less than 12 or 30+ (239,289 exclusions), positive test was not on day of admission or no positive test (20 exclusions). For the analysis of infections of people unvaccinated at positive test, 31 people were were vaccinated at positive test were excluded. For the analysis of infections of people vaccinated at positive test, 150 people were were unvaccinated at positive test were excluded.
Non-participation	Administrative data was used, therefore all individuals whose data were collected in line with the data collection policies for each data source were included.
Randomization	No randomization was applied. The self-controlled case series design means that time-invariant individual characteristics are controlled for. Time-varying factors were controlled for by the inclusion of unexposed cases in the analysis and by the inclusion of calendar time as a covariate.

Reporting for specific materials, systems and methods

We require information from authors about some types of materials, experimental systems and methods used in many studies. Here, indicate whether each material, system or method listed is relevant to your study. If you are not sure if a list item applies to your research, read the appropriate section before selecting a response.

Materials & experimental systems

n/a	Involved in the study	
☒	☐	Antibodies
☒	☐	Eukaryotic cell lines
☒	☐	Palaeontology and archaeology
☒	☐	Animals and other organisms
☒	☐	Clinical data
☒	☐	Dual use research of concern

Methods

n/a	Involved in the study	
☒	☐	ChIP-seq
☒	☐	Flow cytometry
☒	☐	MRI-based neuroimaging
